# The First Annual Report of the Board of the Free Medical and Surgical Dispensing Association of Buffalo

**Published:** 1871-11

**Authors:** 


*The First Annual Report of the Board of Directors of the Free Medical and Surgical Dispensing Association of Buffalo.*

The Report contains the organization and names of the officers, which is supplemented by the Report of the President, containing an account of the objects of the Association and the results to be expected from it.

The expenses of the Association were mainly met by an appropriation of two thousand dollars by the Board of Supervisors of Erie County, this, we infer, being supposed to be the most economical method of caring for the city poor. If it should prove to be the most satisfactory both to the county, the poor and the members of the profession who engage in it, then certainly the plan should be continued and hearty encouragement given it. It will remove the care of the poor from party favoritism, and test the general question, whether the sick can be better cared for by the profession gratuitously, than for very small pay. We rather like the plan as a tax-payer, and only wish its "benevolence"? might be extended to include Poor House, Jail, Penitentiary, and Coroner's accounts. The energy and faithfulness of the profession, in its first years' labor in the Association, merits unqualified admiration.

